# The impact of Scotland's minimum unit pricing for alcohol policy on people accessing services for alcohol dependence: A difference‐in‐difference structured interview study

**DOI:** 10.1111/dar.13960

**Published:** 2024-10-16

**Authors:** Penny Buykx, Andy Perkins, Jane Hughes, Wulf Livingston, Jennifer Boyd, Parvati Perman‐Howe, Allan Johnston, Trevor McCarthy, Alex McLean, Alex Wright, Simon Little, John Holmes

**Affiliations:** ^1^ School of Humanities, Creative Industries and Social Sciences University of Newcastle Newcastle Australia; ^2^ School of Medicine and Population Health University of Sheffield Sheffield United Kingdom; ^3^ Figure 8 Consultancy Services Ltd Dundee United Kingdom; ^4^ Wrexham University Wrexham United Kingdom; ^5^ Salvation Army Centre for Addiction Services and Research, Faculty of Social Sciences University of Stirling Stirling United Kingdom; ^6^ NHS Ayrshire and Arran Kilmarnock United Kingdom

**Keywords:** alcohol, consumption, dependence, policy, price

## Abstract

**Introduction:**

A minimum unit price (MUP) for alcohol of £0.50 per unit (1 UK unit = 10 mL/8 g alcohol) was introduced in Scotland in May 2018. Few previous studies have examined the impact of alcohol pricing policies on people who are alcohol dependent. This study aimed to evaluate the effect of MUP on people who are alcohol dependent including changes in alcohol consumption and health status, as well as potential unintended consequences.

**Methods:**

Three waves of cross‐sectional data were collected in Scotland (intervention) and Northern England (control) at 0–6 months pre‐implementation then 3–9 months and 18–22 months post‐implementation. The sample was *N* = 706 people receiving treatment related to their alcohol use. We collected structured interview data including recent drinking information via a 7‐day timeline‐follow‐back drinking diary. Difference‐in‐difference analyses estimated change in indicators in Scotland compared to England at both post‐implementation timepoints.

**Results:**

The proportion of participants consuming alcohol costing on average <£0.50 per unit in Scotland decreased from 60.6% at 0–6 months prior to MUP implementation to 6.3% at 3–9 months post‐implementation (*p* < 0.0004). There was no significant change in the indicators for alcohol consumption, severity of dependence, health status, other substance use, deprivation level or parenting.

**Discussion and Conclusions:**

The introduction of MUP in Scotland was associated with increases in the prices paid for alcohol by people with dependence and presenting to treatment services. There was no evidence of changes in their alcohol consumption or health status. There was also no evidence of harmful unintended consequences for this population.

**Key Points**

- Evaluation studies suggest the introduction of minimum unit price (MUP) in Scotland reduced alcohol consumption and deaths due to alcohol, including among heavier drinkers and low‐income groups.
- Little is known about the impact of MUP on the subset of heavier drinkers with alcohol dependence. This group may have been affected by unintended consequences, for example, relating to other substance use, health, deprivation and parenting.
- This study shows that in Scotland, people recruited from treatment services with alcohol dependence paid substantially more on average for their alcohol post‐MUP.
- There were no other significant effects of the policy observed for this group in terms of alcohol consumption, severity of dependence, health status, other substance use, deprivation level or parenting indicators.
- This lack of evidence of unintended or detrimental indicators, along with evidence of reductions in alcohol consumption and harms from other sources, is useful information for other jurisdictions considering similar policies.

## 1 INTRODUCTION

Alcohol makes a substantial contribution to the global burden of disease and wider social and economic harms [1, 2, 3]. In the early 2000s, policy makers in Scotland were particularly concerned about high levels of alcohol‐related harm [4]. In 2009 they released a strategic framework outlining a suite of proposed actions intended to reduce consumption and improve early identification and treatment of alcohol problems [4]. A key aspect of the proposed alcohol strategy was to introduce minimum unit pricing (MUP), which sets a floor price for alcohol tied to the number of units of alcohol in the product (1 UK unit = 10 mL/8 g alcohol). This would increase the price of low‐cost, high‐strength alcohol and was anticipated to reduce overall population levels of alcohol consumption, leading to a positive impact on alcohol‐related health and other harms [4]. Although initially legislated for under the *Alcohol (Minimum Pricing) (Scotland) Act* 2012, the policy itself did not come into force until 1 May 2018 due to legal challenges from members of the alcohol industry [5]. When it did so, the MUP threshold was set at £0.50 per unit.

Modelling by the University of Sheffield and evidence from Canada, which has a form of minimum pricing for alcohol, informed policy development in Scotland. Evidence indicated there would be population level reductions in consumption and harms, with benefits concentrated among those who drink more heavily and who are in lower socio‐economic groups [6, 7, 8]. This has since been borne out by evaluation evidence following MUP implementation in Scotland [9, 10, 11] and is consistent with the emerging picture from jurisdictions where comparable policies have been introduced including Canada, Wales, Ireland and the Northern Territory of Australia (e.g., [12, 13]).

However, the modelling undertaken prior to the implementation of MUP in Scotland did not directly examine the potential impact on people who were alcohol dependent. Although this group was not the main target of the policy, there was uncertainty as to how they could be affected. For some, MUP could result in reduction of consumption or severity of alcohol dependence symptoms, contribute to health and wellbeing, or even prevent future cases of alcohol dependence by limiting consumption at an earlier stage. It is also possible, however, that some people may be less able to adjust their consumption in response to increasing prices and so potentially experience unintended consequences [14, 15]. In line with the legislation enabling MUP, a comprehensive evaluation program was carried out by Public Health Scotland. This evaluation strategy drew on a ‘theory of change’ which outlined the expected population level effects of MUP, but also reflected the above concerns and uncertainties, for example, noting that it may have differential effects on spending and substance use for some sub‐groups, for example, substitution of alcohol with other drugs [16].

This paper reports on a study commissioned as part of the overall MUP evaluation program [16] to investigate the impact of the implementation of MUP in Scotland on people who are alcohol dependent. Specifically, this paper aims to examine, among people presenting to services in relation to their alcohol consumption, whether there were changes following the introduction of MUP in key indicators relevant to the abovementioned theory of change. These indicators included effects on alcohol consumption and expenditure, severity of alcohol dependence, other substance use, health status, level of deprivation and parenting.

## 2 METHODS

A detailed description of the study method is available elsewhere [17, 18] and a summary provided below.

### 2.1 Design

Three waves of repeat cross sectional data were collected in two countries; Scotland (where MUP was introduced) and England (where MUP was not introduced). The use of an unexposed comparison site is common practice in public health intervention natural experiment studies in order to help strengthen causal inferences (i.e., the inclusion of a control adds confidence that any observed changes are due to the intervention rather than other factors) [19]. The first wave of data collection occurred 0–6 months prior to MUP implementation (November 2017–April 2018), the second wave 3–9 months post implementation (August 2018–February 2019) and the third wave 18–22 months post implementation (November 2019–March 2020). Structured interviews collected quantitative data from respondents at each wave. The two waves of post‐implementation data collection enabled examination of the stability or otherwise of indicators across a short to mid‐term timeframe. Qualitative interviews were also conducted with a subset of respondents at each wave; however, this paper reports quantitative findings only, with qualitative findings reported elsewhere [20, 21].

### 2.2 Setting

Respondents were recruited from inpatient and community‐based alcohol and drug services, gastroenterology and liver services, and general practices. Services were located in six National Health Service (NHS) areas in Scotland (Glasgow, Edinburgh, Aberdeen, Dumfries and Galloway, the Highlands and Dundee) and four NHS areas in Northern England (Sheffield, Stockport, Newcastle and Liverpool). There were 20 recruitment sites in total: 16 in Scotland and 4 in England, with 1–5 sites per geographic area.

### 2.3 Recruitment

Service providers at each data collection site alerted potentially eligible participants to the study and referred those interested in taking part to the interview team. Eligibility criteria included being over 18 years old, assessed by the service provider as probably alcohol dependent (i.e., have an Alcohol Use Disorders Identification Test score of 16+ [22, 23], or otherwise screened as alcohol dependent by the service), conversant in English and able to provide informed consent.

### 2.4 Data collection

Paper‐based questionnaires were used to collect data at each service. Full details of the data collection instrument are published elsewhere [17]. In brief, the interview tool covered volume and other details of alcohol consumption, including product choice and expenditure for the 7 days prior to entering the service (assessed using the Time Line Follow Back [TLFB] method [24]); alcohol dependence (assessed using the Severity of Alcohol Dependence Questionnaire [25]); other substance use in the past 30 days; health (assessed using the EQ‐5D‐5L [26, 27]); and socio‐demographic questions including household income, main source of income, self‐rating of financial difficulty, and whether or not participants had experienced acute housing problems and/or used foodbanks/charities in the past 3 months. Postcode data were used to determine Index of Multiple Deprivation quintile of residence for Scotland [28] and England [29]. The Index of Multiple Deprivation is an area‐based deprivation measure. Respondents were offered a £10 gift card for a high street retailer in recognition of their time and contribution.

### 2.5 Sample and weighting

The target sample size in Scotland was 200 people per wave and in England 80 per wave. This was a pragmatic decision informed by Scotland being the primary focus of the study and time and resource constraints given study start‐up and recruitment occurred in the less than 6 months between the Scottish Government securing legal permission to introduce MUP and the implementation of the policy. Power calculations indicated that a sample size of 200 per wave in Scotland would be sufficient to detect a 20% reduction in weekly consumption from an anticipated mean consumption of 200 units per week [14]. Wave 3 recruitment concluded early due to the onset of the COVID‐19 pandemic. Across Waves 1 to 3, the achieved sample in Scotland for the structured interviews was 170, 190 and 123 respondents. The corresponding samples sizes for England were 85, 86 and 52 respondents.

The considerations above meant there was substantial variation in the characteristics of the samples collected in each wave (i.e., by sex, age group, geographic region and treatment setting), and therefore we developed weights to improve the comparability of the socio‐demographic characteristics of samples using iterative proportional fitting [30] in R software 3.6.1 (using the pewmethods package [31]). Weights were based on the abovementioned characteristics, with Wave 2 sample characteristics for each country used as the reference sample. This wave was chosen as the reference because it was unaffected by the need to collect data rapidly pre‐implementation (Wave 1) or early cessation of data collection due to the COVID‐19 pandemic (Wave 3).

### 2.6 Outcome indicators

**FIGURE 1:**
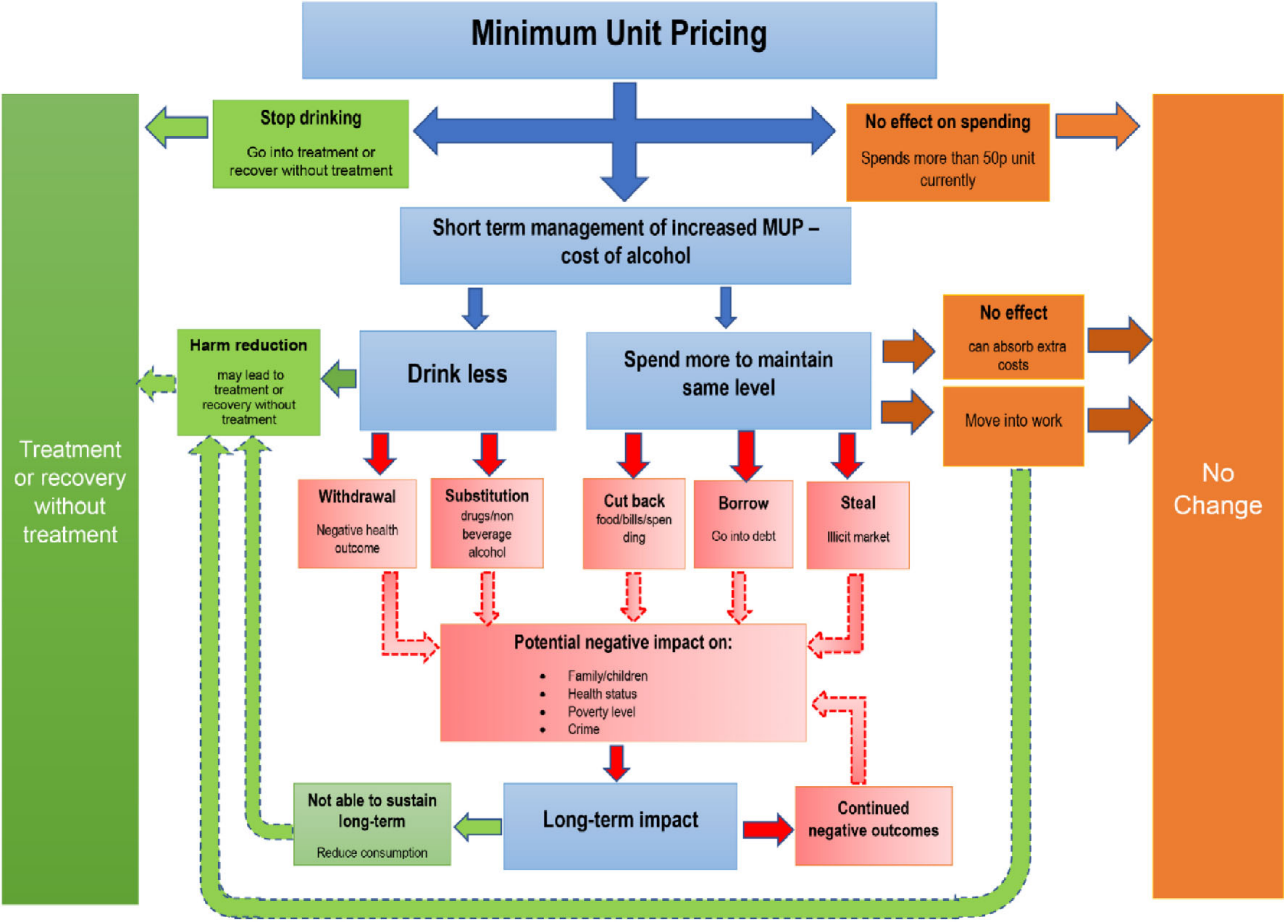
Theory of change for the impact of minimum unit pricing on people drinking at harmful levels [18].

As noted earlier, the theory of change underpinning Public Health Scotland's evaluation strategy was used to guide the choice of indicators, with a particular focus on those most relevant to people drinking at harmful levels [16, 18] (Figure 1). The specific indicators used in each domain (alcohol use and expenditure, alcohol dependence, other substance use, health status, level of deprivation and impact of drinking on parenting) were discussed and agreed prior to analysis with our evaluation advisory group (Table 1).

**TABLE 1:** Domain of interest and specific measures.

Domain indicator	Specific measures
Alcohol use in the TLFB week	Alcohol units consumed (mean, SD) Alcohol expenditure (mean, SD) Price paid per unit of alcohol (mean, SD) % individuals whose first drink of TLFB week cost <£0.50 per unit % individuals who on average paid <£0.50 per unit % individuals who consumed from each product category (e.g., cider <7.5% ABV, wine, etc)
Alcohol dependence	SADQ scores (mean, SD) % individuals in each SADQ dependence category (mild, moderate, severe)
Other substance use (last 30 days)	% individuals using any illicit substance (excluding illicitly obtained benzodiazepines, antidepressants or painkillers) % individuals using illicitly obtained benzodiazepines, antidepressants or painkillers % individuals using prescribed benzodiazepines, antidepressants or painkillers
Health status	Self‐rating of health (0–100) on EQ‐5D‐5L (mean, SD) % individuals scoring 4–5 in each of five EQ‐5D‐5L health domains
Deprivation	% individuals reporting: Household income <£300 per week Benefits as main source of income Living in most deprived IMD quintile Finding it ‘quite’ or ‘very’ difficult to manage financially Acute housing problem in past 3 months Foodbank/charity use in past 3 months
Parenting	% individuals with dependent children reporting negative impact of drinking on: How they have felt about their parenting Getting children to school or appointments Children having treats Children having to act more grown up

*Note*: EQ‐5D‐5L, A standardised instrument measuring quality of life across five domains (mobility, self‐care, usual activities, pain/discomfort and anxiety/depression) and a visual analogue scale where respondents rate their health today from 0 to 100.

Abbreviations: ABV, alcohol by volume; IMD, Index of Multiple Deprivation; SADQ, Severity of Alcohol Dependence Questionnaire (scores range from 0 to 60 with <16 indicating low dependency, 16 to 30 indicating moderate dependency and 31 to 60 indicating severe dependency); SD, standard deviation; TLFB, time line follow back.

### 2.7 Analysis

Difference‐in‐difference analyses was conducted to compare changes in indicator variables over time in Scotland versus England. Analyses compared changes between Wave 1 and Wave 2 and between Wave 1 and Wave 3, with wave, country and the interaction between wave and country used as predictor variables. The results report on the key parameter of interest, which was the interaction between wave and country, to indicate whether there was a significant difference in changes observed in Scotland compared to changes observed in England over the same period. Logistic, ordinal and linear regression models were specified for binary, ordered and continuous variables respectively. The distribution of the mean alcohol units consumed in the TLFB week and mean total alcohol expenditure in the TLFB week variables were positively skewed so were log‐transformed. A Bonferroni adjustment was made to the conventional *p*‐value threshold of *p* = 0.05 to account for running multiple tests, for a revised threshold of *p* = 0.0004630 [32].

### 2.8 Ethics

The study was approved by the NHS Scotland (West of Scotland) Research Ethics Committee 3 (dated 01/09/2017).

## 3 RESULTS

### 3.1 Sample characteristics and subgroups

Weighted respondent characteristics and the type of service they were recruited from by country and wave are shown in Table 2. The characteristics of the sample were in line with typical treatment populations, with a higher proportion of males and those in middle‐age. Most had an Alcohol Use Disorders Identification Test score of 20+ and a large minority of participants had dependent children. After weighting, the Scottish sample had a higher proportion of men, was older, and less likely to have dependent children compared to the English sample. The proportion of respondents recruited from inpatient alcohol and drug settings increased in Scotland across waves; all respondents in England were recruited from community/outpatient settings. The vast majority of respondents identified as White (data not shown due to small cell sizes).

**TABLE 2:** Participant characteristics, AUDIT score, service type/setting, qualitative interviews and weighting by country and wave.

	Scotland Wave 1		Scotland Wave 2		Scotland Wave 3		England Wave 1		England Wave 2		England Wave 3	
	*N* = 170	% (w)^a^	*N* = 190	%	*N* = 123	% (w)^a^	*N* = 85	% (w)^b^	*N* = 86	%	*N* = 52	% (w)^b^
Sex												
Male	118	66.7	128	67.4	80	66.4	61	58.1	50	58.1	35	58.4
Female	52	33.3	62	32.6	43	33.6	24	41.9	36	41.9	17	41.6
Age group, years												
≤29	11	5.5	10	5.3	3	3.1	10	13.0	11	12.8	3	5.2
30–39	37	17.0	33	17.4	28	20.0	19	21.9	19	22.1	19	29.8
40–49	47	31.3	61	32.1	33	31.9	28	29.1	25	29.1	15	29.2
50–59	59	35.9	54	28.4	39	26.9	23	29.4	21	24.4	13	30.5
60+	16	10.3	32	16.8	20	18.0	5	6.6	10	11.6	2	5.3
Has dependent children												
Yes	44	25.8	46	24.2	44	34.3	35	40.8	36	41.9	25	46.6
Service type and setting												
Alcohol and drug	126	82.6	154	81.1	107	80.6	81	89.5	77	89.5	47	89.5
Community/outpatient	98	63.9	74	38.9	43	41.6	81	89.5	77	89.5	47	89.5
Inpatient	28	18.7	80	42.1	64	39.1	0	0.0	0	0.0	0	0.0
Gastroenterology or liver	36	14.2	33	17.4	16	19.4	4	10.5	9	10.5	5	10.5
Community/outpatient	8	3.1	12	6.3	0	0.0	4	10.5	9	10.5	5	10.5
Inpatient	28	11.1	21	11.1	16	19.4	0	0.0	0	0.0	0	0.0
General practitioner	8	3.2	3	1.6	0	0.0	0	0.0	0	0.0	0	0.0
Scotland												
Glasgow	70	49.3	92	48.4	80	50.6						
Edinburgh (Lothian)	39	18.9	35	18.4	25	19.1						
Aberdeen (Grampian)	30	16.2	30	15.8	6	14.4						
Dumfries and Galloway	18	2.7	16	4.7	7	4.8						
Highlands	11	8.6	8	8.4	1	8.6						
Dundee (Tayside)	2	4.3	9	4.2	4	2.5						
England												
Sheffield							36	29.1	25	29.1	8	28.8
Stockport (Pennines)							20	18.6	16	18.6	5	18.6
Newcastle (Northumberland)							17	2.4	21	24.4	19	24.5
Liverpool							12	27.9	24	27.9	20	28.0

Abbreviation: AUDIT, Alcohol Use Disorders Identification Test.

^a^ Weighted with reference to Scotland Wave 2.

^b^ Weighted with reference to England Wave 2.

### 3.2 Alcohol use

#### 3.2.1 Consumption

Alcohol consumption during the TLFB week varied substantially across waves in both countries with no clear trend and wide variation between individuals (Table 3). In Scotland, mean units in the TLFB week fell from 187.5 at Wave 1 to 168.0 at Wave 2, then rose back to 192.0 at Wave 3. There was no significant difference between Scotland and England in the change in the mean number of units consumed by respondents from Wave 1 to 2 or from Waves 1 to 3.

**TABLE 3:** Alcohol consumption, alcohol expenditure and product consumed (over the TLFB week), by country and wave.

	Scotland			England			Wave 1–Wave 2			Wave 1–Wave 3		
	Wave 1	Wave 2	Wave 3	Wave 1	Wave 2	Wave 3	Exp β	SE	*p*‐value	Exp β	SE	*p*‐value
Alcohol consumption												
Alcohol units (mean)^a^	187.5	168.0	192.0	167.9	147.4	179.9	0.06	0.07	0.423	−0.01	0.08	0.950
Alcohol units (SD)^a^	(132.1)	(121.5)	(142.1)	(107.0)	(112.8)	(134.1)	‐	‐	‐	‐	‐	‐
Alcohol expenditure												
Spending £ (mean)^a^	82.6	95.2	106.9	77.3	68.7	89.9	0.15	0.07	0.032	0.07	0.08	0.376
Spending £ (SD)^a^	(59.4)	(60.6)	(76.8)	(49.0)	(51.4)	(64.7)	‐	‐	‐	‐	‐	‐
Price paid per unit £ (mean)	0.49	0.60	0.59	0.50	0.59	0.55	0.09	0.04	0.011	0.07	0.04	0.054
Price paid per unit £ (SD)	(0.25)	(0.18)	(0.19)	(0.20)	(0.33)	(0.21)	‐	‐	‐	‐	‐	‐
1st drink TLFB <£0.50 per unit (%)	56.2	12.1	19.5	53.3	43.0	33.0	**−0.17**	**0.41**	<0.0004*	−0.42	0.46	0.061
Average price <£0.50 per unit (%)	60.6	6.3	14.4	54.1	45.2	32.2	**0.06**	**0.47**	<0.0004*	0.27	0.49	0.008
Product consumed (%)												
Cider <7.5% ABV	20.8	21.1	10.6	17.1	19.8	6.2	0.85	0.47	0.736	1.42	0.73	0.633
Cider ≥7.5% ABV	25.0	9.5	6.7	19.4	12.8	8.0	0.52	0.52	0.204	0.60	0.71	0.470
Beer <7.5% ABV	38.7	30.0	38.3	41.2	39.5	31.6	0.73	0.38	0.412	1.49	0.44	0.366
Beer ≥7.5% ABV	7.9	3.7	2.2	7.9	3.5	4.2	1.05	0.86	0.952	0.50	1.05	0.513
Vodka	33.0	34.7	35.6	32.0	26.7	33.3	1.39	0.40	0.411	1.06	0.06	0.896
Wine	14.9	22.1	28.4	26.4	37.2	26.1	0.98	0.43	0.967	2.30	0.50	0.094
Whisky	14.5	7.9	4.2	11.1	2.3	9.0	2.65	0.87	0.262	0.33	0.78	0.151
Tonic wine	5.3	7.9	7.1	0.0	1.2	0.0	0.00	4.4E3	0.997	1.20	7E3	1.000
Other^b^	6.7	10.0	13.6	16.6	15.1	5.2	1.74	0.57	0.336	7.99	0.80	0.009

*Note*: β, coefficient of intervention effect parameter in difference‐in difference model; *p*‐values are the significance of the intervention effect parameter for difference‐in‐difference models comparing wave 1 to wave 2 and wave 3 separately, * and bold font indicates *p*‐value is significant at our Bonferroni corrected threshold of *p* < 0.0004630.

Abbreviations: ABV, alcohol by volume; SD, standard deviation; SE, standard error; TLFB, Time Line Follow Back.

^a^ Measure logged for analysis.

^b^ “Other” alcohol included cocktails, liqueurs, vermouth, schnapps, rum, brandy and tequila.

#### 3.2.2 Expenditure

The average price paid per unit of alcohol by respondents in Scotland was £0.49 before MUP was implemented (Wave 1), £0.60 following implementation (Wave 2) and £0.59 at Wave 3 (Table 3). However, price changes were also evident in England, and thus the difference‐in‐difference between countries did not reach significance. Similarly, although there was a rising trend in mean total alcohol expenditure in Scotland in the TLFB week across waves, this was not significantly different to the trend in expenditure in England over the same period.

Between Waves 1 and 2, there was a significantly greater reduction in Scotland than England in the proportion of respondents who on average paid less than £0.50 per unit in the TLFB week (Scotland: 60.6% to 6.3%, England: 54.1% to 45.2%, w1‐w2: *p* < 0.0004) (Table 3). The difference between countries in the decrease observed from Waves 1 and 3 approached but did not reach significance. Likewise, between Waves 1 and 2 there was a significantly greater reduction in Scotland than England in the proportion of respondents reporting that their first drink of the TLFB week cost less than £0.50 per unit (Scotland: 56.2% to 12.1%, England: 53.3% to 43%, w1‐w2: *p* < 0.0004), but this was not significant between Waves 1 and 3.

#### 3.2.3 Products consumed

Following the introduction of MUP in Scotland, the proportion of respondents consuming high strength cider and high strength beer (≥7.5% alcohol by volume [ABV]) in that country followed a marked decreasing trend across waves (Table 3). However, these changes were not significant due to similar declines in England. The apparent decline in high strength cider and beer consumption in Scotland did not appear to be offset by an increase in vodka consumption, which remained stable. There was an upward trend in the proportion of respondents in Scotland consuming wine from Wave 1 to Wave 3, in contrast to relative stability in England, however, this difference was not significant.

### 3.3 Alcohol dependence

There was no significant change in mean Severity of Alcohol Dependence Questionnaire scores (measured on a scale of 0–60) or categorisation as mild, moderately or severely dependent following the introduction of MUP in Scotland compared to the same period in England (Table 4).

**TABLE 4:** Alcohol dependence, other substance use (in the 30 days prior to interview) and health status (measured using EQ‐5D‐5L) by country and wave.

	Scotland			England			Wave 1–Wave 2			Wave 1–Wave 3		
	Wave 1	Wave 2	Wave 3	Wave 1	Wave 2	Wave 3	Exp β	SE	*p*‐value	Exp β	SE	*p*‐value
Alcohol dependence												
SADQ score (mean)	39.4	36.1	37.3	29.5	30.1	37.3	−3.96	2.94	0.178	−2.74	−3.36	0.415
SADQ score (SD)	(14.0)	(16.8)	(18.2)	(15.5)	(16.0)	(14.3)	‐	‐	‐	‐	‐	‐
Mild (SADQ 0‐15) (%)	10.8	16.0	17.6	21.4	24.4	16.4	0.59	0.37	0.108	0.59	0.42	0.164
Moderate (SADQ 16‐30) (%)	15.3	22.5	14.1	33.0	27.9	32.6	‐	‐	‐	‐	‐	‐
Severe (SADQ 31‐60) (%)	74.0	61.5	68.3	44.8	47.7	51.1	‐	‐	‐	‐	‐	‐
Other substance use (%)												
Prescribed substances^a^	63.7	62.1	55.1	72.3	60.5	66.2	1.59	0.39	0.237	0.93	0.45	0.877
Illicitly obtained prescribed substances	14.9	13.2	9.8	2.5	10.5	2.9	0.19	0.84	0.046	0.53	1.15	0.580
Other illicit substances	30.9	22.1	24.1	25.4	26.7	26.8	0.59	0.42	0.214	0.66	0.48	0.386
Tobacco	30.9	36.3	26.3	40.7	44.2	34.7	1.05	0.37	0.792	1.25	0.44	0.951
Health status by domain (%)^b^												
Anxiety/depression	28.2	36.3	35.8	36.7	37.2	46.0	1.42	0.39	0.368	0.97	0.44	0.938
Pain/discomfort	18.9	22.6	22.1	24.3	23.3	17.7	1.33	0.44	0.517	1.83	0.54	0.260
Mobility	18.9	16.8	12.5	12.3	8.1	7.9	1.38	0.58	0.585	1.01	0.71	0.989
Usual activities	16.6	16.9	17.2	14.8	11.6	12.5	0.30	0.54	0.576	1.27	0.61	0.696
Self‐care	7.4	6.3	10.5	3.8	2.3	0.9	1.41	1.00	0.735	6.61	1.67	0.259
Self‐rating of health (0‐100)^c^												
Self‐rating (mean)	50.3	49.4	48.2	54.7	56.1	56.1	−2.31	4.20	0.582	−3.45	4.71	0.465
Self‐rating (SD)	(21.7)	(22.8)	(21.7)	(23.2)	(23.3)	(22.1)	‐	‐	‐	‐	‐	‐

*Note*: β, coefficient of intervention effect parameter in difference‐in difference model; *p*‐values are the significance of the intervention effect parameter for difference‐in‐difference models comparing wave 1 to wave 2 and wave 3 separately.

Abbreviations: SADQ, Severity of Alcohol Dependence Questionnaire; SD, standard deviation; SE, standard error.

^a^ Prescribed substances include benzodiazepines, antidepressants or painkillers.

^b^ EQ‐5D‐5L: score of 4 (severe problems) or 5 (extreme problems).

^c^ EQ‐5D‐5L Visual Analogue Scale: 0 = worse health you can imagine, 100 = best health you can imagine.

### 3.4 Other substance use

There were no significant changes in the use of other substances in the 30 days prior to interview following the introduction of MUP in Scotland compared to the same period in England (Table 4). Overall, in both countries and across all waves, the most used other substances were prescribed benzodiazepines, antidepressants or painkillers (prevalence ranging from 55.1% to 72.3%), followed by tobacco (26.3–44.2%); illicit substances (22.1–30.9%) and illicitly obtained prescribed substances (2.5–14.9%).

### 3.5 Health status

There were no significant changes in any of the health status indicators in Scotland following the introduction of MUP compared to the same period in England (Table 4). In both countries, the most common health problem to be experienced at a severe or extreme level was anxiety/depression (28.2–46.0%), followed by pain/discomfort (17.7–24.3%). Mean health ratings were consistent across waves.

### 3.6 Deprivation

There were no significant changes in any of the deprivation indicators in Scotland following the introduction of MUP compared to the same period in England (Table 5).

**TABLE 5:** Deprivation and impact of drinking on parenting by country and wave.

	Scotland			England			Wave 1–Wave 2			Wave 1–Wave 3		
	Wave 1	Wave 2	Wave 3	Wave 1	Wave 2	Wave 3	Exp β	SE	*p*‐value	Exp β	SE	*p*‐value
Experience of deprivation (%)												
Low household income^a^	82.3	75.8	68.2	64.4	57.0	51.6	0.92	0.41	0.834	0.78	0.45	0.585
Benefits are main income	75.7	66.8	62.6	44.9	55.8	55.4	0.42	0.39	0.024	0.35	0.44	0.017
Lowest IMD quintile^b^	37.3	33.2	31.8	46.5	46.5	45.1	0.84	0.38	0.633	0.83	0.43	0.673
Struggling financially^c^	32.1	35.3	38.4	31.4	38.4	29.8	0.85	0.39	0.672	1.42	0.46	0.439
Foodbank or charity use	22.7	17.9	22.3	13.1	19.8	25.8	0.46	0.50	0.113	0.42	0.53	0.108
Acute housing problems	9.1	10.5	14.8	9.9	18.6	20.2	0.56	0.58	0.318	0.75	0.62	0.643
Negative impact of drinking on parenting (%)^d^												
Feelings about parenting	17.3	16.8	22.0	13.8	19.8	24.6	0.63	0.50	0.348	0.66	0.54	0.439
Children having to act more grown up	9.9	11.1	13.1	5.2	8.1	5.4	0.70	0.72	0.616	1.33	0.87	0.744
Getting children to school/appointments	3.4	9.5	10.3	4.4	7.0	1.8	1.82	0.84	0.474	8.15	1.28	0.100
Children having treats	5.6	8.9	9.7	6.7	9.3	1.8	1.15	0.71	0.839	7.01	1.21	0.109

*Note*: β, coefficient of intervention effect parameter in difference‐in difference model; *p*‐values are the significance of the intervention effect parameter for difference‐in‐difference models comparing wave 1 to wave 2 and wave 3 separately.

Abbreviations: IMD, Index of Multiple Deprivation; SE, standard error.

^a^ Household income less than £300 per week.

^b^ Live in most deprived Index of Multiple Deprivation quintile for Scotland or England.

^c^ Finding it quite or very difficult to manage financially.

^d^ Data from respondents with dependent children only.

### 3.7 Parenting

Among respondents who had dependent children, there were no significant changes in self‐reported negative impacts of alcohol consumption on parenting in Scotland following the introduction of MUP compared to the same period in England (Table 5). The most common concern in both countries was respondents' feelings about their parenting (13.8%–24.6%).

## 4 DISCUSSION

The results above provide no evidence that the introduction of MUP in Scotland in May 2018 had an impact on alcohol consumption levels reported by people in Scotland using services in relation to alcohol dependence. These results should be interpreted in the context of the wider evaluation program for MUP, which examines a wide range of populations and outcomes using different but complementary datasets and methodologies to form an overall conclusion on the effects of the policy [33]. The results of the present study contrast with evidence reported by Wyper et al [11] of a significant decrease in deaths from liver disease immediately following the introduction of MUP, particularly among lower income groups, along with decreased hospitalisations for alcoholic liver disease and alcohol psychoses. Those decreases may plausibly arise from changes in drinking among people with alcohol dependence, as well as among those in the wider population who drink at higher levels, who are reported to have reduced their consumption [10]. The inconsistency between our findings and those of Wyper et al. [11] may also reflect the limitations of the present study for detecting modest changes in alcohol consumption among those who present to treatment services.

Our findings indicate MUP affected expenditure per unit of alcohol, with a significant reduction in the proportion of respondents purchasing alcohol below the threshold price after 3–9 months. However, there was no significant increase in overall expenditure or average price paid per unit due to broadly similar changes occurring in both England and Scotland. These results are consistent with reports of generally good compliance by alcohol retailers with the requirement to apply a minimum price to alcohol products [34]. Some alcohol purchasing was reported under the £0.50 per unit threshold post‐implementation by a minority (<15%) of participants. While this is most likely due to minor reporting errors in the TLFB, there may have also been some limited instances of under‐the‐counter selling, as reported elsewhere [21, 35]. There was less consumption of strong cider (≥7.5% ABV) in Scotland and England, which is consistent with the policy intention of MUP to target low cost, high strength products and aligns with wider evidence that this market is declining [36]. There is some evidence that the UK‐wide policy debate around MUP, as well as its implementation in Scotland, is prompting action by producers and retailers that is contributing to this decline [37, 38]. Indeed, the producer of one high strength cider brand started selling a reformulated version in Scotland following MUP‐implementation, with the ABV reduced from 7.5% to 6% [39].

Our study found no evidence of an effect of MUP among people accessing treatment with alcohol dependence on any other indicators, including those associated with potential unintended and negative consequences, such as substance use, economic deprivation or parenting behaviours. This lack of evidence of detrimental outcomes is an important finding, given earlier concerns about the impact of the policy for this group [14, 15]. The quantitative data reported in this paper are largely consistent with qualitative accounts of the impact of MUP from the same participant group reported elsewhere [18, 21] and from other studies within the evaluation program [40, 41]. Those qualitative accounts, however, indicated some people experienced financial strain, which our quantitative measures of deprivation (e.g., self‐reports of struggling financially, use of foodbanks or charities, acute housing problems) did not detect, suggesting they may be modest or, in line with the qualitative findings, limited to those in the most economically vulnerable circumstances. Our findings are also consistent with the lack of evidence for a change in prescriptions for alcohol dependence following the introduction of MUP, although the data used to examine changes in prescriptions had limitations that are discussed elsewhere [42].

This study has several strengths. It is one of the first studies to evaluate quantitatively the impact of a major alcohol pricing intervention on those with alcohol dependence. Detailed information was collected regarding recent alcohol consumption, expenditure, and other key variables from a relatively hard to reach population regarding a key policy implementation question. Data were collected pre‐intervention and at two post‐intervention time points in Scotland and at control sites in England. It can be challenging to recruit people who are alcohol dependent, and we had a limited timeframe in which to do so. It is therefore a strength of the study that we were able to recruit to within 15% of our target for Waves 1 and 2 (with Wave 3 recruitment unavoidably curtailed due to the COVID‐19 pandemic). Weighting was applied by sex, age group, geographic region and treatment setting to account for unavoidable differences in the sample structure at each wave.

A key limitation of the study is that it uses repeat cross‐sectional data, thus individual patterns of drinking and expenditure were not tracked over time. This was necessary due to the likely challenges in retaining people in a longitudinal study over time and in disentangling the effects of MUP from those of treatment. As a consequence, the characteristics of those with dependence, those presenting to treatment, and those recruited to the sample may vary across waves in ways that do not reflect the impact of MUP. Additionally, the introduction of MUP in Scotland may have encouraged particular sub‐groups of people to attend treatment who would not have otherwise done so. Weighting the sample to match sex, age, geographic region and treatment setting over time, including a control population, and exploring multiple indicators to allow understanding of more complex effects all sought to address this concern. Further, given the costs associated with recruiting community samples of people with alcohol dependence, the study only included people attending treatment settings: therefore, the findings may not be generalisable to those not in contact with services, which includes the majority of people with alcohol dependence. Similarly, those people attending services who were not referred to the study by staff at the recruitment sites, or who chose not to participate, may also differ from those who took part. Although data were collected at three separate time points (rather than asking participants to recall their drinking over the entire time period of the evaluation), respondents were asked to recall detailed drinking information for an entire week and there may have been some inaccuracies. To mitigate potential issues with recall, interviewers experienced in working with people with dependence guided respondents through the data collection instrument and visual aids (e.g., pictures of common alcohol products) were used.

Future research regarding the impact of MUP and other pricing policies among people who are alcohol dependent could usefully explore experiences of financial strain, particularly in the context of the current cost‐of‐living crisis. Likewise, investigation of health indicators over the longer term is needed to understand the extent to which the reduction in alcohol‐attributable deaths found by Wyper et al. [11] following the introduction of MUP reflect reductions in deaths among those with and without dependence.

## 5 CONCLUSIONS

The introduction of MUP in Scotland was associated with increases in the prices paid for alcohol by people with dependence and presenting to treatment services. There was no evidence of changes in their alcohol consumption or health status. There was also no evidence of harmful unintended consequences for this population.

## AUTHOR CONTRIBUTIONS

Each author certifies that their contribution to this work meets the standards of the International Committee of Medical Journal Editors.

## CONFLICT OF INTEREST STATEMENT

Penny Buykx has been involved in the evaluation of MUP in the Northern Territory of Australia, and Andy Perkins and Wulf Livingston have been involved in the evaluation of MUP in Wales. John Holmes has played a substantial role in producing, disseminating and debating key evidence that informed the introduction of MUP in Scotland and has played a similar role in other jurisdictions in the United Kingdom and internationally.

## Data Availability

Author elects to not share data.
